# High Modulation Depth Enabled by Mo_2_Ti_2_C_3_T_x_ MXene for Q-Switched Pulse Generation in a Mid-Infrared Fiber Laser

**DOI:** 10.3390/nano12081343

**Published:** 2022-04-13

**Authors:** Xin Guo, Shuai Wang, Peiguang Yan, Jinzhang Wang, Linpeng Yu, Wenjun Liu, Zhijian Zheng, Chunyu Guo, Shuangchen Ruan

**Affiliations:** 1 Shenzhen Key Laboratory of Laser Engineering, Guangdong Provincial Key Laboratory of Micro/Nano Optomechatronics Engineering, Key Laboratory of Optoelectronic Devices and Systems of Ministry of Education and Guangdong Province, College of Physics and Optoelectronic Engineering, Shenzhen University, Shenzhen 518060, China; xguo@szu.edu.cn (X.G.); wangshuai2019@email.szu.edu.cn (S.W.); yanpg@szu.edu.cn (P.Y.); jzwang@szu.edu.cn (J.W.); pyu@szu.edu.cn (L.Y.); scruan@sztu.edu.cn (S.R.); 2 Key Laboratory of Advanced Optical Precision Manufacturing Technology of Guangdong Higher Education Institutes, Shenzhen Technology University, Shenzhen 518118, China; 3 State Key Laboratory of Information Photonics and Optical Communications, School of Science, Beijing University of Posts and Telecommunications, Beijing 100876, China; jungliu@bupt.edu.cn; 4 Shenzhen Institute of Information Technology, Shenzhen 518172, China; zhengzhijian87@foxmail.com

**Keywords:** MXenes, double transition metal carbides, saturable absorber, mid-infrared laser, nonlinear optics

## Abstract

Two-dimensional (2D) materials show great promise as saturable absorbers (SAs) for ultrafast fiber lasers. However, the relatively low modulation depth and poor stability of some 2D materials, such as graphene and black phosphorus, restrict their applications in the mid-infrared pulse generation. Herein, we first report a novel 2D double transition metal carbide, denoted as Mo_2_Ti_2_C_3_T_x_ MXene, as the saturable absorber (SA) for a passively Q-switched mid-infrared fiber laser. Due to the unique four-metal atomic layer structure, the Mo_2_Ti_2_C_3_T_x_ exhibits superior saturable absorption properties, particularly with a higher modulation depth (40% at 2796 nm) than most of the other reported 2D SA materials. After incorporating the MXene SA with an erbium-doped fiber system, the passively Q-switched pulses were achieved with a repetition rate of 157.3 kHz, the shortest pulse width of 370 ns, and single-pulse energy of 1.92 μJ, respectively. Such results extend the MXene-based SAs as promising candidates for advanced photonic devices.

## 1. Introduction

Pulsed mid-infrared fiber lasers have diverse applications in the fields of spectroscopy, gas sensing, medical surgery, and free-space communication [1,2]. Passively mode-locking or Q-switching technology relying on saturable absorbers (SAs) is one of the most effective ways to achieve pulsed lasers because of its merits in terms of compact structure, simplicity, and cost-efficiency. A variety of SA materials have been researched for mid-infrared pulse generation, including the semiconductor saturable absorber mirror (SESAM), dye- or ion-doped crystals, and metal nanoparticles [3,4,5,6,7]. However, most of these SAs have their own shortcomings that fail to satisfy the SA requirements for practical mid-infrared fiber lasers. For example, although SESAMs is the most mature choice of SAs available, its complicated fabrication process, high cost, inflexibility, and narrow wavelength range (cannot cover the mid-infrared region beyond 3.1 μm yet) severely limit its widespread applications. Therefore, it is imperative to develop novel functional materials as SAs for the mid-infrared pulsed fiber lasers.

Two-dimensional (2D) materials have attracted extensive research attention for pulse generation owing to their unique advantages, such as broad spectral and ultrafast response, tunable band gap, low fabrication, and integration costs [8,9,10,11,12]. However, further explorations are still demanded to overcome some challenges, including low modulation depth, poor stability, and the lack of a well-controlled synthetic method [13,14]. Recently, a new family of 2D transition metal carbides and/or nitrides, known as MXenes, have been thrust into the limelight due to their distinctive chemical and physical properties [15,16,17]. MXenes share a general formula of M_n+1_X_n_T_z_ (*n* = 1–4), where “M” represents one/mixed transition metals, “X” represents carbon and/or nitrogen, and “T_z_” represents the surface functional group terminations (such as –O, –OH, –F, and –Cl). As the biggest 2D family, MXenes possess the merits of adjustable chemical compositions and surface terminations, high electrical conductivity, good hydrophilicity, and superior mechanical and optical properties, which enable a great number of applications [18].

Particularly, several pioneering works have demonstrated the excellent nonlinear optical absorption performance of MXenes as broadband SAs. Jhon et al. first realized femtosecond laser pulses centralized at 1557 nm from fiber cavities by using Ti_3_CNT_x_ MXene as a mode-locker [19]. The 2D structure and the electronic properties of MXene are well conserved, regardless of the stacking, due to the unique interlayer coupling formed by the surface terminations. Subsequently, many MXene-based SAs have been reported for pulse generation, covering several different types MXenes (Ti_3_C_2_, Ti_2_C, V_2_C, Nb_2_C, Mo_2_C, etc.), as well as a wide range of wavebands from near- to mid-infrared regions [20,21,22,23,24,25]. In addition, the advantages of MXenes’ saturable absorption properties, such as large modulation depths and high damage thresholds, compared to other 2D materials, are well verified and recognized. Nevertheless, the research on MXene-based SAs for mid-infrared lasers is currently restricted to the Ti_3_C_2_T_x_, the most common MXene composition, and further explorations on other types of MXenes are urgently required to exploit their potentialities in this field [26,27,28].

Herein, for the first time, we synthesized and applied few-layer Mo_2_Ti_2_C_3_T_x_ MXene, a double transition metal carbide material with the four atomic metal layer structure, as the saturable absorber for a passively Q-switched mid-infrared fiber laser. The nonlinear saturable absorption behavior of Mo_2_Ti_2_C_3_T_x_ was investigated at 2796 nm using a homemade power-dependent system. Notably, an enhanced modulation depth of 40% was observed within the double transition carbide compared to previously reported MXenes. By using the Mo_2_Ti_2_C_3_T_x_ MXene SA, we acquired passively Q-switched laser pulses with a repetition rate, shortest pulse width, and single-pulse energy of 157.3 kHz, 370 ns, and 1.92 μJ, respectively. The results demonstrate the great potential of double transition metal carbides for mid-infrared pulse modulation and generation and expand the MXenes’ application in the optical devices.

## 2. Mo_2_Ti_2_C_3_T_x_ MXene Synthesis and Characterization

The Mo_2_Ti_2_C_3_T_x_ was prepared according to the previously reported top-down method [29]. Typically, 1 g of Mo_2_Ti_2_AlC_3_ powder was slowly added to 10 mL of 48% HF concentrated solution in a polytetrafluoroethylene (PTFE) vial. The mixed solution was stirred with a PTFE magnetic bar at 55 °C for 96 h, while a lid was loosely placed back on the vial to prevent the evaporation of the solution. The resultant suspension was washed with distilled water several times until the pH of the supernatant reached 6–7. Then, the supernatant was decanted and 10 mL of tetrabutylammonium hydroxide (35 wt.% TBAOH in water) aqueous solution was added into the obtained sediment, which was kept stirring for 24 h at room temperature subsequently. After that, the redundant TBAOH was washed away with DI water three times, and 50 mL of water was added to the intercalated MXene sediment. Finally, the mixture was sonicated for 1 h under the protection of Ar gas and centrifuged for 30 min, and the few-layer Mo_2_Ti_2_C_3_T_x_ colloidal solution (dark brown color, Figure S1a) was achieved for further use. Meanwhile, the few-layer Ti_3_C_2_T_x_ nanoflakes were also prepared as a reference sample using a similar wet-chemical method (see details in Supporting Information). The Ti_3_C_2_T_x_ colloidal solution displays a dark blue color due to the different chemical composition (Figure S1b).

**Figure 1:**
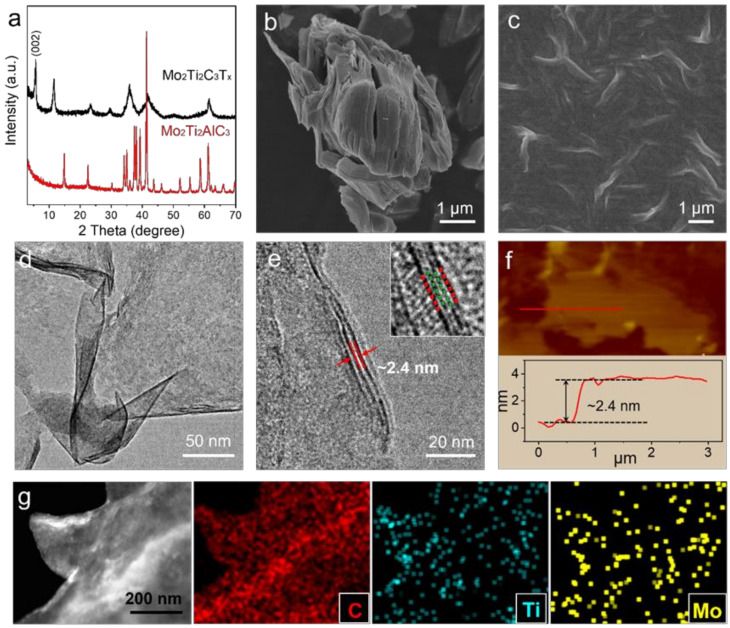
(**a**) XRD patterns of the Mo_2_Ti_2_AlC_3_ precursor and Mo_2_Ti_2_C_3_T_x_ MXene materials. SEM images of (**b**) etched multilayer and (**c**) exfoliated few-layer Mo_2_Ti_2_C_3_T_x_ nanosheets. (**d**) TEM and (**e**) HRTEM images of the few-layer Mo_2_Ti_2_C_3_T_x_ nanosheets. (**f**) AFM image of the Mo_2_Ti_2_C_3_T_x_ nanosheets and the corresponding height profile. (**g**) STEM image and EDS mapping results of the C, Ti, and Mo elements.

The as-prepared materials were characterized by X-ray diffraction (XRD, Rigaku D/MAX 2200), and the results in Figure 1a show that all characteristic peaks of Mo_2_Ti_2_AlC_3_ are replaced by the (00l) peaks belonging to Mo_2_Ti_2_C_3_T_x_. The shift of the (002) peak toward a lower angle (2 theta ~5.68 degree) indicates the increase of interlayer spacing due to the presence of intercalated water or protons. The scanning electron microscopy (SEM, FEI Scios02) image of the etched Mo_2_Ti_2_C_3_T_x_ displays an accordion-like morphology, which is different from the plate-like bulks of the Mo_2_Ti_2_AlC_3_ (Figure S2). The SEM and transition electron microscopy (TEM, FEI Tecnai G2 F30) images of the exfoliated Mo_2_Ti_2_C_3_T_x_ exhibit a typical crumpled 2D morphology, demonstrating the success of the exfoliation process (Figure 1c,d). Similar nanosheets morphology was observed in the SEM image of the few-layer Ti_3_C_2_T_x_ (Figure S3). The high-resolution TEM (HRTEM) image indicates that the few-layer Mo_2_Ti_2_C_3_T_x_ nanosheets have an enlarged interlayer spacing of 2.4 nm. Additionally, the four transition metal layers with the ABBA structure can be clearly identified in the magnified HRTEM image (inset of Figure 1e).

The atomic force microscopy (AFM, Bruker D3100) measurement was performed to observe the topography of the exfoliated Mo_2_Ti_2_C_3_T_x_ (Figure 1f). The corresponding scanning profile shows that the thickness of monolayer MXene is around 2.4 nm, which is consistent with the HRTEM result. The scanning transmission electron microscopy (STEM) image and corresponding energy dispersive spectrum (EDS) elemental mapping images of Ti, Mo, and C further confirm the chemical compositions of Mo_2_Ti_2_C_3_T_x_ (Figure 1g).

The few-layer MXene nanosheets were layer-by-layer deposited uniformly on a commercial gold mirror by a spray-coating method for optical properties measurement and pulse generation. Typically, the exfoliated MXene colloidal solution (0.5 mg mL^−1^) was sprayed onto an oxygen plasma-treated gold mirror by an airbrush (Mr. Airbrush Procon Boy PS-289). The nozzle size was 0.3 mm, and the operating pressure was controlled at about 60 psi. The outlet of the airbrush was maintained perpendicular to the surface of gold mirror with a distance of 25 cm. The deposition area was isolated by paper tapes, and airflow was employed to dry the film after each coating. Finally, a Mo_2_Ti_2_C_3_T_x_- and Ti_3_C_2_T_x_-coated gold mirror was obtained for optical property measurement and application (Figure S4).

**Figure 2:**
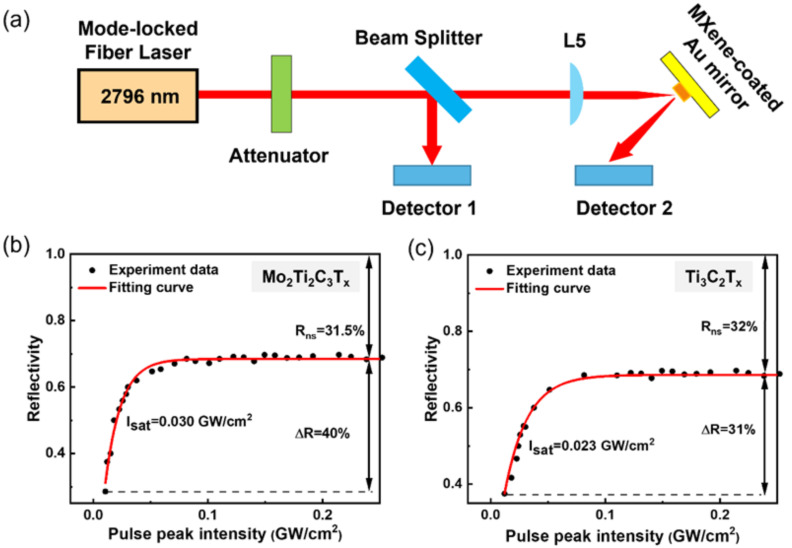
(**a**) The experimental setup for the nonlinear saturable absorption characteristics measurement at 2796 nm. The reflectivity of the (**b**) Mo_2_Ti_2_C_3_T_x_ and (**c**) Ti_3_C_2_T_x_ as a function of peak pulse intensity.

The nonlinear saturable absorption characteristics of Mo_2_Ti_2_C_3_T_x_ MXene were measured using a typical power-dependent setup, as presented in Figure 2a. The laser source is a homemade NPR mode-locked fiber laser centralizing at 2796 nm with a pulse width of 600 fs and a repetition rate of 52 MHz. A bandpass filter (LP1600) was used to remove the remaining pump light. The optical loss was composed of two polarizers and a half-wave plate. The output power of the second polarizer could be changed by adjusting the angle of the half-wave plate. A broadband plate beam splitter (Thorlab, BSW510, USA) with a 50:50 ratio was placed at an angle of 45° with respect to the light path to separate the laser source into two beams.

The reflected beam was used as the reference power recorded by a power meter detector (Detector 1). The transmitted beam was focused on the surface of the Mo_2_Ti_2_C_3_T_x_-coated Au mirror, which was slightly tilted to reflect the beam, and enabled it to reach another power meter detector (Detector 2). A series of reflectivity could be obtained by adjusting the angle of the half-wave plate to change the incident laser power. Figure 2b shows the curve of reflectivity at different pulse peak powers, and the experimental data were fitted according to the following formula:

(1) $$R\left(I\right)=1-\Delta R\;*\;\exp\left(-I/I_{\mathrm{sat}}\right)-R_{\mathrm{ns}}$$

where R(I) is the reflectivity, ΔR is the modulation depth, I is the input pulse energy, I_sat_ is the saturation energy, and R_ns_ is the non-saturable loss. As shown in Figure 2b, the computed modulation depth, saturable power, and non-saturation loss of the Mo_2_Ti_2_C_3_T_x_ are 40%, 0.03 GW cm^−2^, and 31.5%, respectively.

By contrast, the Ti_3_C_2_T_x_ sample displays a much lower modulation depth of 31% and saturable power of 0.023 GW cm^−2^ under the same testing condition (Figure 2c), which is in good agreement with the previously reported values [30]. The enhanced modulation depth and saturable power of the Mo_2_Ti_2_C_3_T_x_ could be ascribed to its unique four-atomic-metal layer structure and the strong interactions between MXene layers via the connection of hydrogen bonds on the surface terminations.

## 3. Mid-Infrared Lasers Modulated by Mo_2_Ti_2_C_3_T_x_ MXene

**Figure 3:**
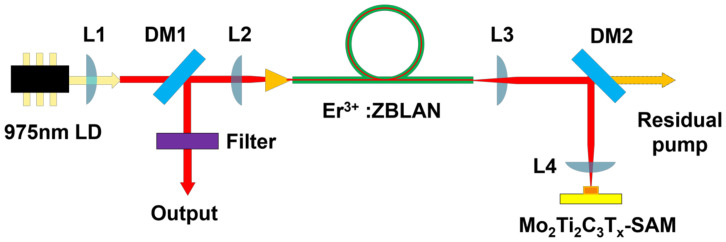
Schematic of the experimental setup for the passively Q-switched Er^3+^-doped ZBLAN fiber laser.

In the experiment, a 2.8 μm Q-switched erbium-doped fluoride fiber laser was built by using the Mo_2_Ti_2_C_3_T_x_-coated Au mirror, as shown in Figure 3. Specifically, the pump source is a commercial 30 W fiber-coupled laser diode with a core diameter of 105 μm and a numerical aperture (NA) of 0.22. A plano-convex NBK-7 lens with a focal length of 15 mm (L1) and a plano-convex CaF2 lens with a focal length of 20 mm (L2) was used to collimate and focus the pump light into the gain fiber. A 1.6 m long double-cladding 7 mol% Er^3+^: ZBLAN fiber (LVF, France) was used as the gain media, which has the core diameter of 15 μm (NA = 0.12) and the inner circular cladding diameter of 260 μm (NA > 0.4) broken by two parallel flats separated by 240 μm. The front end of the gain fiber was perpendicularly cleaved to provide 4% Fresnel reflection, and the other end of the optical fiber was cleaved at an 8° angle to avoid parasitic oscillation. Finally, the 4% Fresnel reflection of the front end of the gain fiber and the reflection of Mo_2_Ti_2_C_3_T_x_ saturable absorber mirror (SAM) formed a laser resonant cavity. Considering that the V parameter of the fiber is 2.5 at 2.8 μm, the signal laser was operating in a single mode. A dichroic mirror (T = 85% at 976 nm, R = 98% at 2800 nm) was placed at 45° between the two lenses to output the signal laser.

Two plano-convex CaF2 lenses with a focal length of 15 mm (L3 and L4) were employed to collimate and focus the signal laser onto Mo_2_Ti_2_C_3_T_x_-SAM. Another dichroic mirror (DM2) removed the remaining pump laser to avoid thermal damage to MXene-SAM. A long-pass filter (LP1600) was placed at the output of the laser to filter out the residual 976 nm pump light. The average output power of the laser was measured with a power meter (Throlab PM100D). The pulse train was monitored by a mid-infrared detector (PCI-9, 250 MHz bandwidth, VIGO) connected with a 1 GHz bandwidth digital oscilloscope (MSO4032, Tektronix, USA). The radio frequency spectrum and optical spectrum of the output laser were monitored by a spectrum analyzer (N9320A, Agilent) and an optical spectrum analyzer (Yokogawa AQ6375), respectively.

**Figure 4:**
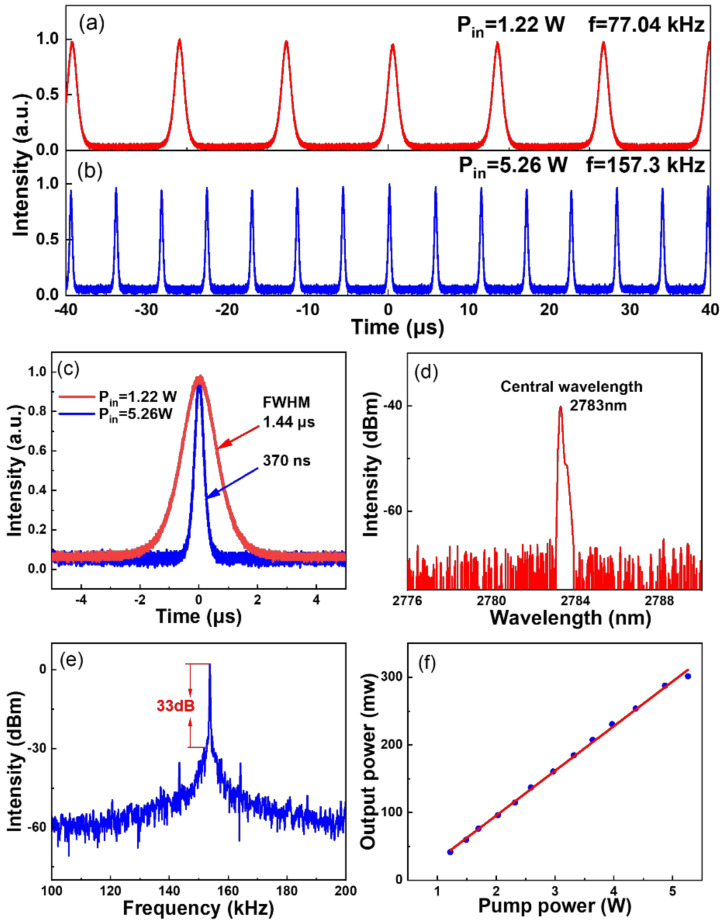
Q-switched pulse trains using the Mo_2_Ti_2_C_3_T_x_-SAM at the pump power of (**a**) 1.22 W and (**b**) 5.26 W, and (**c**) the corresponding single-pulse waveforms. (**d**) The optical spectrum of the Q-switched pulses and (**e**) the measured radio frequency at the pump power of 5.26 W. (**f**) The linear relationship between pump power and output power.

The continuous-wave (CW) threshold value of the fiber laser was 0.43 W. An unstable Q-switched pulse train was first observed with the pump power increased to 0.97 W. As the pump power was increased to 1.22 W, stable Q-switched pulse trains were generated with a repetition frequency of 77.04 kHz and a pulse width of 1.44 μs (Figure 4a). All pulse widths are calculated based on the full width at half maximum (FWHM). The Q-switched pulse trains remained stable as the pump power was continuously increased to 5.26 W, where the pulse showed the highest repetition frequency of 157.3 kHz and a minimum pulse width of 370 ns (Figure 4b,c).

Figure 4d,e illustrate the optical and frequency spectrum of the Q-switched pulses at the pump power of 5.26 W. The center wavelength of the pulse spectrum was 2783 nm, and the signal-to-noise (SNR) of fundamental frequency was 34 dB, which attested that the Q-switched pulse was stable. Figure 4f shows the relationship between the output power and the incident pump power. As the incident pump power grew from 1.22 W to 5.26 W, the average output power was increased from 42 mW to 301 mW. As a comparison, although Q-switched pulses were obtained by using the Ti_3_C_2_T_x_-SAM, the operation was stable at a narrower power range and showed the highest repetition frequency of 131.29 kHz and a minimum pulse width of 770 ns, respectively (Figure S5).

The pulse became unstable again when the pump power was further increased, and completely reverted to CW operation when the pump power exceeded 6.25 W. This pulse instability can be ascribed to the excessive heat accumulation under the strong laser signal. It was worth noting that the Q-switched state could be recovered when the power was reduced to 6.05 W, demonstrating the superior structural stability of the Mo_2_Ti_2_C_3_T_x_ MXene-SAM.

**Figure 5:**
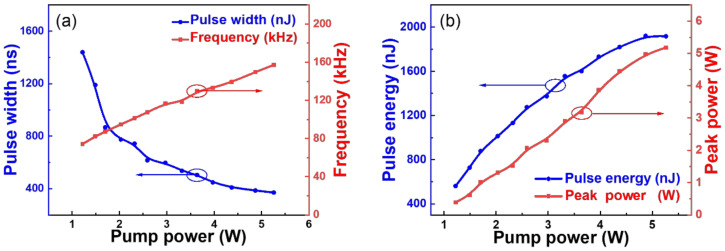
(**a**) Repetition frequency and pulse width as functions of the pump power. (**b**) Peak power and pulse energy as functions of the pump power.

Figure 5a depicts the repetition frequency and pulse width as a function of the incident pump power. The repetition frequency was increased from 77.04 kHz to 157.3 kHz as the pump power rose from 1.22 to 5.26 W. Meanwhile, the pulse width was decreased from 1.44 µs to 370 ns, conversely. The phenomenon complies with the typical features of Q-switched operation. Figure 5b displays the calculated pulse energy and pulse peak power under different pump powers; the obtained maximum pulse energy and peak power are 1.92 μJ and 5.18 W, respectively. By contrast, the derived maximum pule energy and peak power of the Ti_3_C_2_T_x_-SAM modulated pulse trains are 1.80 μJ and 4.2 W, respectively (Figure S6).

To further evaluate the potential of 2D double transition metal carbide (Mo_2_Ti_2_C_3_T_x_) for mid-infrared fiber laser generation, we compare the modulation depth of several reported SAs and the output performance of Q-switched fiber lasers based on these SAs (Table 1). The results show that Mo_2_Ti_2_C_3_T_x_ MXene has the highest modulation depth, and we obtained the narrowest pulse width of the 2.8 µm passive Q-switched fluoride fiber lasers. The significantly improved modulation depth and structural stability can be attributed to the unique double atomic transition metal layer structure of Mo_2_Ti_2_C_3_T_x_ with less exposed metal atoms compared to the previously investigated MXenes (such as Ti_3_C_2_T_x_, Ti_3_CNT_x_, and Ti_2_CT_x_).

**Table 1:** Comparison of several reported passive Q-switched erbium-doped fluoride fiber lasers based on nanomaterial saturable absorbers.

SA Types	Wavelength (nm)	Repetition Rate (kHz)	Pulse Width (μs)	Modulation Depth	Ref.
Sb	2799.7	58.8	1.7	n/a	[31]
BP	2779	63	1.18	15%	[12]
Graphene	2783	37	1.0	n/a	[7]
Gold nanostars	2800	125	0.534	25%	[32]
PtSe_2_	2865	238.1	0.62	39.57%	[33]
InSe	2765	54	1.2	12%	[34]
Ti_3_C_2_T_x_ MXene	2786.2	78.12	1.04	33.2%	[30]
Mo_2_Ti_2_C_3_T_x_	2783	157.3	0.37	40%	This work

## 4. Conclusions

In conclusion, we first reported a passively Q-switched mid-infrared fiber laser by using the 2D double transition metal carbide (Mo_2_Ti_2_C_3_T_x_) as SAM. The maximum pulse energy of 1.92 μJ at a repetition rate of 157.3 kHz was achieved. The experimental results demonstrate that the Mo_2_Ti_2_C_3_T_x_ with a narrow band gap, high modulation depth, and excellent structural stability is conducive to pulse shortening. The significantly enhanced nonlinear optics can be assigned to the unique multi-metal atomic layer structure of Mo_2_Ti_2_C_3_T_x_. This work greatly expands the MXene frontiers for laser generation, and it may open a door for developing and engineering 2D materials-based saturable absorbers for advanced photonic devices.

## Data Availability

The date presented in this study are available on request from the authors.

## Supplementary Materials

The following supporting information can be downloaded at: https://www.mdpi.com/article/10.3390/nano12081343/s1, Figure S1: Digital images of the aqueous colloidal solution of the few-layer (a) Mo_2_Ti_2_C_3_T_x_ and (b) Ti_3_C_2_T_x_ materials; Figure S2: SEM image of the Mo_2_Ti_2_AlC_3_ material; Figure S3: SEM image of the few-layer Ti_3_C_2_T_x_ material; Figure S4: The digital photo of the Mo_2_Ti_2_C_3_T_x_ and Ti_3_C_2_T_x_ deposited on the gold mirror by a spray-coating method; Figures S5 and S6: Characteristics of the Ti_3_C_2_T_x_-SAM modulated Q-switched mid-infrared pulses.
