# Supplementary material for: High Modulation Depth Enabled by Mo2Ti2C3Tx MXene for Q-Switched Pulse Generation in a Mid-Infrared Fiber Laser
Source: Nanomaterials (Basel). 2022 Apr 13;12(8):1343. doi: 10.3390/nano12081343 (PMC9025076; doi:10.3390/nano12081343)
Supplement: Supplementary file 1 [file nanomaterials-12-01343-s001.zip › nanomaterials-1649598-supplementary.pdf]

nanomaterials

Supplementary Material

High Modulation Depth Enabled by $Mo_2Ti_2C_3T_x$ MXene for Q-Switched Pulse Generation in a Mid-Infrared Fiber Laser

Xin Guo [1], Shuai Wang [1,2], Peiguang, Yan [1], Jinzhang Wang [1], Linpeng, Yu [1], Wenjun Liu [3], Zhijian Zheng [4], Chunyu Guo [1,*] and Shuangchen Ruan [1,2]

1 Shenzhen Key Laboratory of Laser Engineering, Guangdong Provincial Key Laboratory of Micro/Nano Opto-mechatronics Engineering, Key Laboratory of Optoelectronic Devices and Systems of Ministry of Education and Guangdong Province, College of Physics and Optoelectronic Engineering, Shenzhen University, Shenzhen 518060, China; xguo@szu.edu.cn (X.G.); wangshuai2019@email.szu.edu.cn (S.W.); yanpg@szu.edu.cn (P.Y.); jzwang@szu.edu.cn (J.W.); pyu@szu.edu.cn (L.Y.); scruan@sztu.edu.cn (S.R.)
2 Key Laboratory of Advanced Optical Precision Manufacturing Technology of Guangdong Higher Education Institutes, Shenzhen Technology University, Shenzhen 518118, China
3 State Key Laboratory of Information Photonics and Optical Communications, School of Science, Beijing University of Posts and Telecommunications, Beijing 100876, China; jungliu@bupt.edu.cn
4 Shenzhen Institute of Information Technology, Shenzhen 518172, China; zhengzhijian87@foxmail.com
* Correspondence: cyguo@szu.edu.cn

Supplementary Experimental Details *Synthesis of few-layer $Ti_3C_2T_x$ material*. $Ti_3C_2T_x$ nanosheets were prepared through a top-down wet chemical route based on the previous report. Typically, 1 g of MAX (Ti_3AlC_2) was slowly added into the mixed solution consisting of lithium fluoride and hydrochloric acid (1 g of LiF in 10 mL of 9 mol g^{-1} of HCl). After reacting at 35 °C for 24 h, the solid product was washed with deoxygenated water until the pH of the supernatant reached 6~7. The last solid product was mixed with 250 ml of water and sonicated under the protection of Ar atmosphere for 1 h. Finally, the mixture was centrifuged at 3500 rpm for 1 h and the supernatant was kept as few-layer $Ti_3C_2T_x$ colloidal solution.

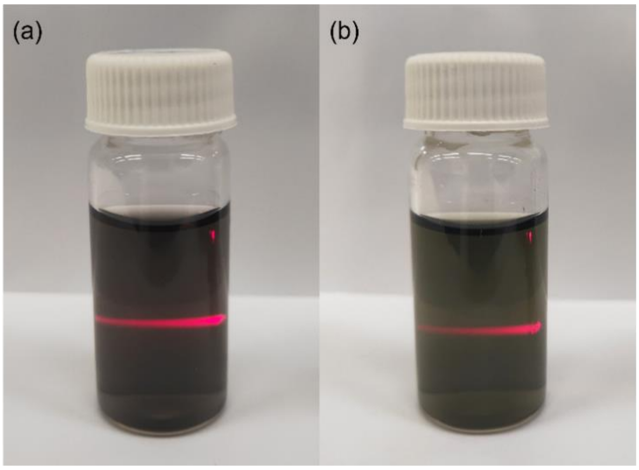

Figure S1. Digital images of the aqueous colloidal solution of the few-layer (**a**) $Mo_2Ti_2C_3T_x$ and (**b**) $Ti_3C_2T_x$ materials. Red light beams were incident from the side to demonstrate the Tyndall effect.

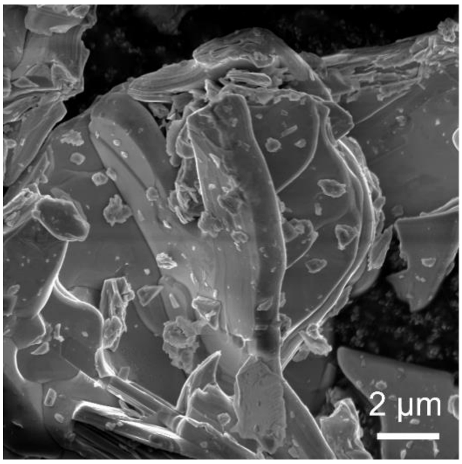

Figure S2. SEM image of the $Mo_2Ti_2AlC_3$ material.

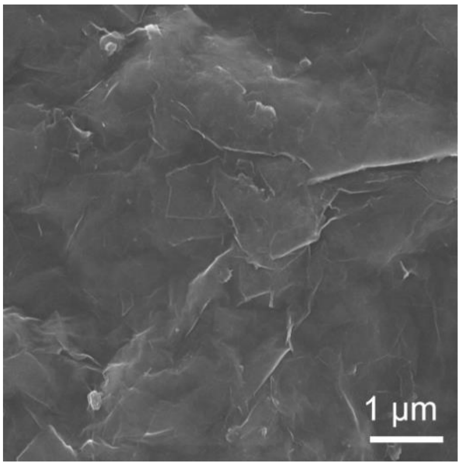

Figure S3. SEM image of the few-layer $Ti_3C_2T_x$ material.

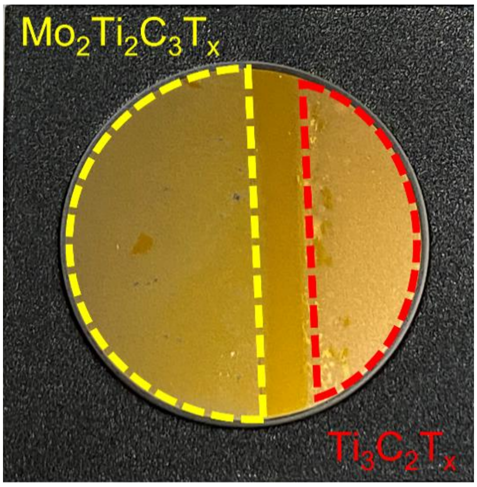

Figure S4. The digital photo of the $Mo_2Ti_2C_3T_x$ and $Ti_3C_2T_x$ deposited on the gold mirror by a spray-coating method.

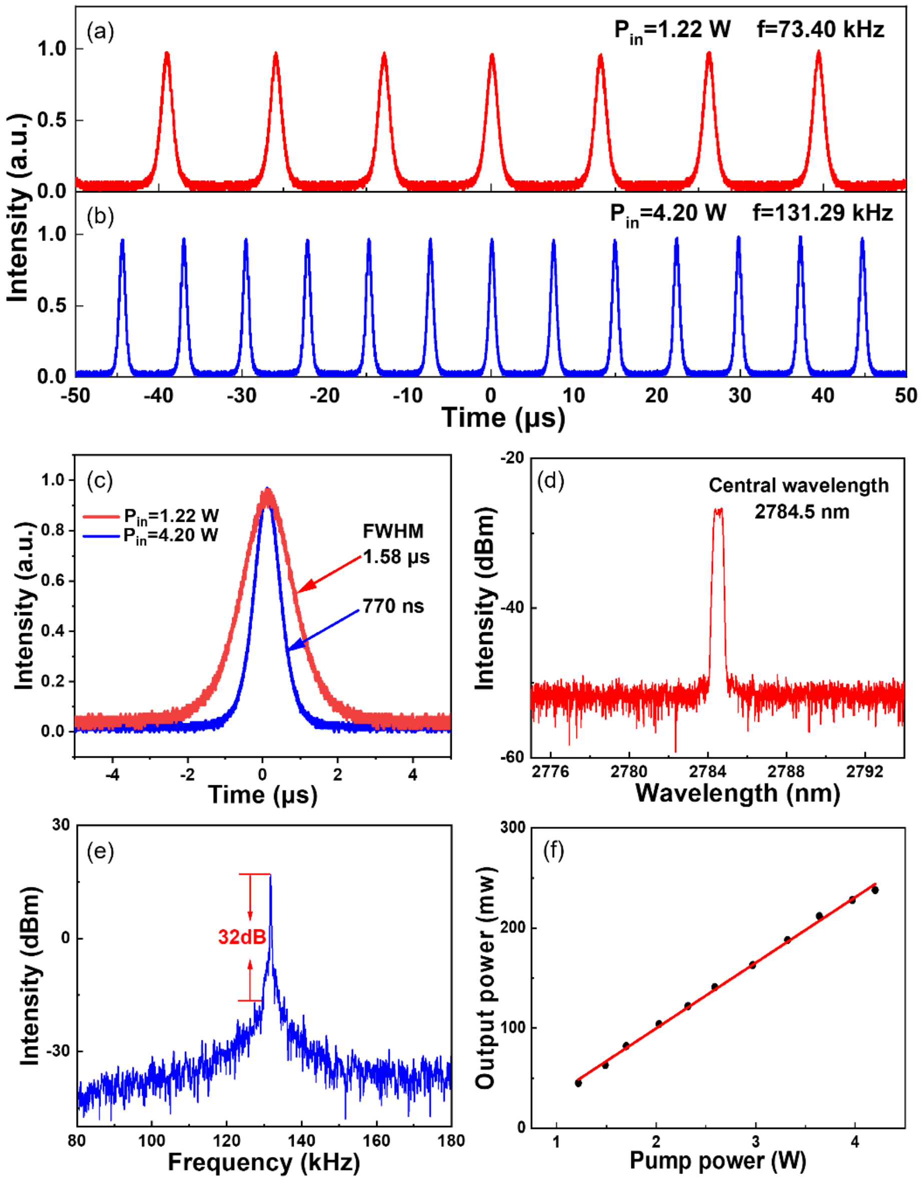

Figure S5. Q-switched pulse trains using the $Ti_3C_2T_x$-SAM at the pump power of (**a**) 1.22 W and (**b**) 4.20 W, and (**c**) the corresponding single-pulse waveforms. (**d**) The optical spectrum of the Q-switched pulses and (**e**) the measured radio frequency at the pump power of 4.20 W. (**f**) The linear relationship between pump power and output power.

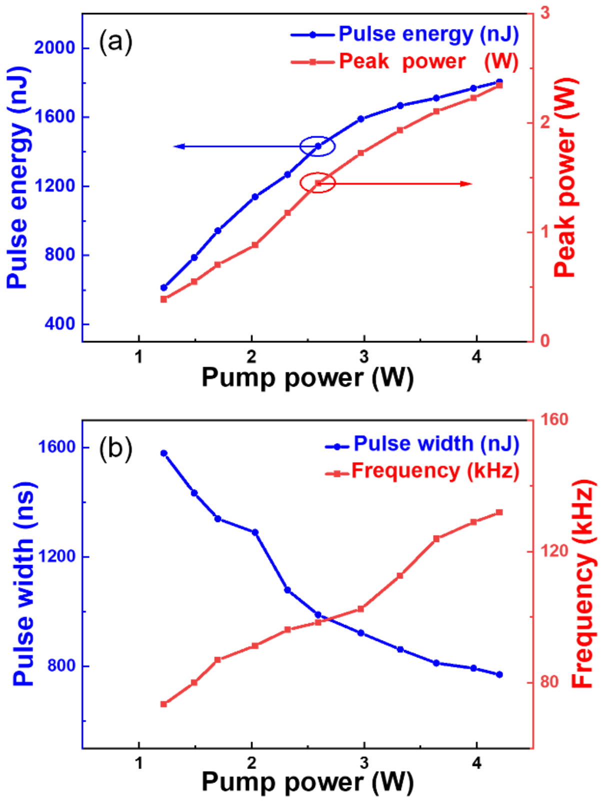

Figure S6. Characteristics of the Q-switched pules using $Ti_3C_2T_x$-SAM. (**a**) Repetition frequency and pulse width as functions of the pump power. (**b**) Peak power and pulse energy as functions of the pump power.
